# Statins Do Not Alter the Incidence of Mesothelioma in Asbestos Exposed Mice or Humans

**DOI:** 10.1371/journal.pone.0103025

**Published:** 2014-08-05

**Authors:** Cleo Robinson, Helman Alfonso, Samantha Woo, Amy Walsh, Nola Olsen, Arthur W. Musk, Bruce W. S. Robinson, Anna K. Nowak, Richard A. Lake

**Affiliations:** 1 National Centre for Asbestos Related Diseases, School of Medicine and Pharmacology, University of Western Australia, Harry Perkins Institute for Medical Research, Nedlands, Perth, Western Australia, Australia; 2 Anatomical Pathology, PathWest, Perth, Western Australia, Australia; 3 School of Public Health, Curtin University, Perth, Western Australia, Australia; 4 Occupational Respiratory Epidemiology, School of Population Health, University of Western Australia, Perth, Western Australia, Australia; 5 Department of Respiratory Medicine, Sir Charles Gairdner Hospital, Perth, Western Australia, Australia; Virginia Commonwealth University, United States of America

## Abstract

Mesothelioma is principally caused by asbestos and may be preventable because there is a long latent period between exposure and disease development. The most at-risk are a relatively well-defined population who were exposed as a consequence of their occupations. Although preventative agents investigated so far have not been promising, discovery of such an agent would have a significant benefit world-wide on healthcare costs and personal suffering. Statins are widely used for management of hypercholesterolemia and cardiovascular risk; they can induce apoptosis in mesothelioma cells and epidemiological data has linked their use to a lower incidence of cancer. We hypothesised that statins would inhibit the development of asbestos-induced mesothelioma in mice and humans. An autochthonous murine model of asbestos-induced mesothelioma was used to test this by providing atorvastatin daily in the feed at 100 mg/kg, 200 mg/kg and 400 mg/kg. Continuous administration of atorvastatin did not alter the rate of disease development nor increase the length of time that mice survived. Latency to first symptoms of disease and disease progression were also unaffected. In a parallel study, the relationship between the use of statins and development of mesothelioma was investigated in asbestos-exposed humans. In a cohort of 1,738 asbestos exposed people living or working at a crocidolite mine site in Wittenoom, Western Australia, individuals who reported use of statins did not have a lower incidence of mesothelioma (HR = 1.01; 95% CI = 0.44–2.29, p = 0.99). Some individuals reported use of both statins and non-steroidal anti-inflammatory drugs or COX-2 inhibitors, and these people also did not have an altered risk of mesothelioma development (HR = 1.01; 95% CI = 0.61–1.67, p = 0.97). We conclude that statins do not moderate the rate of development of mesothelioma in either a mouse model or a human cohort exposed to asbestos.

## Introduction

Mesothelioma is a cancer of the pleural and peritoneal cavities, caused predominantly by asbestos exposure [1]. Following its widespread use in multiple industries, millions of people are living with known asbestos exposure and preventative measures against mesothelioma would have a global impact. Whilst asbestos use and mining have been banned in many countries, they are still permitted in some regions and safe disposal of the asbestos already in use remains a considerable hazard [2]. Thus, mesothelioma is an ongoing concern and its incidence continues to rise in industrialized and developing nations: asbestos related diseases are predicted to remain a substantial economic and human burden across the world for the foreseeable future [1].

Epidemiological data indicating beneficial correlations between cancer incidence and dietary intake of vitamins, minerals and commonly used drugs has intensified interest in finding cancer prevention strategies and instigated many randomised controlled trials and animal studies [3]–[6]. An understanding of cancer mechanisms and biological activities of such compounds has supported their potential for cancer prevention. Although mesothelioma does not usually feature in the epidemiological studies because of its relative rarity, it is a good candidate for targeted prevention because the carcinogen is known, and the ‘at risk’ population can be readily identified.

Asbestos triggers chronic inflammation and production of reactive oxygen and nitrogen species. These are both carcinogenic mechanisms that are recognised hallmarks of cancer which could be targeted by preventative agents to inhibit mesothelioma development [7]. The long-term persistence of asbestos fibres lodged within the body and the long latency between asbestos exposure and disease development suggests that an agent that is safe for long-term use would be appropriate. Anti-oxidants and anti-inflammatories have been investigated for their ability to prevent cancer in different models with a range of effectiveness, from none, to a substantial reduction in cancer development [8].

Despite a strong rationale, the antioxidants, vitamins A, E, D and selenium were found to be ineffective in preventing mesothelioma [9], [10]. This is consistent with the finding that in humans beta carotene and retinoic acid forms of vitamin A do not affect mesothelioma incidence [11]. We have also investigated the use of aspirin and cyclooxygenase-2 inhibitors (COX-2i), again finding no benefit in either the mouse model or a human cohort [12], [13]. Here the search for a prevention strategy was extended to investigate the ability of statins to inhibit mesothelioma development in asbestos exposed mouse and human populations.

Statins inhibit the enzyme 3-hydroxy-3-methylglutaryl coenzyme A (HMG-CoA) reductase, which is key to the production of endogenous cholesterol, a fundamental structural component of cell membranes and essential for cell proliferation. Statins are widely used, often with long term regularity and at a known dosage, thus enabling large epidemiological studies. These studies have suggested an association between statin use and a reduced incidence of cancers including lung, breast, prostate, colon and bowel [14]. However, this finding conflicts with a meta-analysis of 27 randomised controlled trials that did not show protective effects [15]. Animal models and cell line studies demonstrate the role of statins in many cellular mechanisms (proliferation, apoptosis, angiogenesis, cell migration and inflammation), supporting the rationale for its preventative action on cancer growth or development [16]–[18]. In mesothelioma, lovastatin induces apoptosis in vitro [19] and was effective in vivo when combined with doxorubicin chemotherapy, possibly by reversing doxorubicin resistance of the cells [20].

A combination of chemoprevention agents could be more effective by repression of a wider range of the mechanisms involved in tumorigenesis. Furthermore, this strategy may also result in improved efficacy of safer and lower doses of drugs such as aspirin and statins, which have potential toxicities associated with long-term use.

In this study the effect of high, medium and low doses of statin on the survival rate of asbestos-induced mesothelioma is investigated in the MexTAg mouse model of asbestos-induced mesothelioma, which is highly suited to testing potential cancer prevention agents [12]. Alongside this investigation human epidemiological data has been analysed from the Wittenoom cohort, consisting of people who lived or worked in Wittenoom while asbestos mining was taking place. Participants were recruited from a cancer surveillance project for which significant asbestos exposure was the key inclusion criterion [21]. Participants reported use of statins at a defined time point in the recruitment questionnaire and were followed up until 2011. We hypothesised that use of statins would be associated with lower incidence of mesothelioma, and that the combined use of both non-steroidal anti-inflammatory drugs (NSAIDs) or COX-2i and statins would have an additional benefit.

## Materials and Methods

### Transgenic mice

MexTAg 299h transgenic mice were generated by insertion of a 2,148-bp of SV40 TAg open reading frame cloned downstream of 1,850 bp of the mesothelin promoter as described previously [22]. Groups of experimental mice were matched for age and gender.

### Asbestos-induced mesothelioma

Asbestos fibres (IUCC reference sample of Wittenoom Gorge crocidolite, Western Australia) were suspended in PBS (6 mg/ml) and passed through a 23-gauge needle several times. MexTAg 299 h mice were injected in the peritoneum with two doses of 3 mg asbestos, 1 month apart. Mice were monitored and the animals were euthanized when ascites became evident or sickness, distress or loss of condition was noticed (endpoint). A standardized-system of criteria was used to define the first signs of disease and this was taken as the diagnosis date [12]. All experiments had University of Western Australia Animal Ethics Committee approval (#RA5/100/1075) and were carried out in strict accordance with National Health and Medical Research Council guidelines.

### Animal diets

Diets were made by Specialty Feeds, Glen Forest, Western Australia. The base diet was AIN93 and was used for control groups. Atorvastatin was added to AIN93 at 100, 200 and 400 mg/kg at source and provided ad libitum.

### Human study population

Former workers and residents of Wittenoom have been followed up since 1975. In 1989 all ex-residents and/or ex workers were invited to participate in the “Vitamin A programme” which began accepting participants in July 1990 [23]. On acceptance for the trial participants completed a questionnaire, which included their current use of medication. The statins reported as used were simvastatin, atorvastatin, pravastatin, rosuvastatin or fluvastatin. There was no indication of dosage, frequency of use or duration of use. Out of 2,363 participants in the Vitamin A programme, 1,738 provided information on medication use.

Incident cases of cancer were obtained from the West Australian Cancer Registry, the Western Australia Mesothelioma Registry, and the Registrar General's Office for WA. The date of an incident cancer was taken as the first relevant diagnostic pathological specimen. Participants were followed up until diagnosis of mesothelioma or death, or were censored at 30^th^ June 2012. There were 107 mesothelioma cases during this follow up period.

## Statistical Analysis

### Animal studies

Kaplan Meier survival curves were compared by log rank test for survival. The one-way ANOVA test for variance was used to analyse latency time and survival time data from three or more test groups. The non-parametric, unpaired, two-tailed t test was used to compare data from two test groups.

### Human cohort

Baseline descriptive data were shown as mean and standard deviations (SD), or percentages (%). On average participants were followed up for 11.94 years (SD 6.28, IQR = 6.67–17.80). Cox regression modelling was applied to examine the association (as hazard ratios and their 95% CI) between each main exposure (statins, alone or in combination with COX-2i or NSAIDs) and mesothelioma. Analyses were adjusted for age, sex, body mass index, smoking and intensity of asbestos exposure. The assumption of proportionality was examined by plotting the Schoenfeld residuals. Alpha was set at 5% and all statistical tests were two-tailed. The statistical package Stata version 12.1 (StataCorp, College Station, Texas, USA) was used.

## Results

### Statins do not alter survival of asbestos-induced mesothelioma

**Figure 1:**
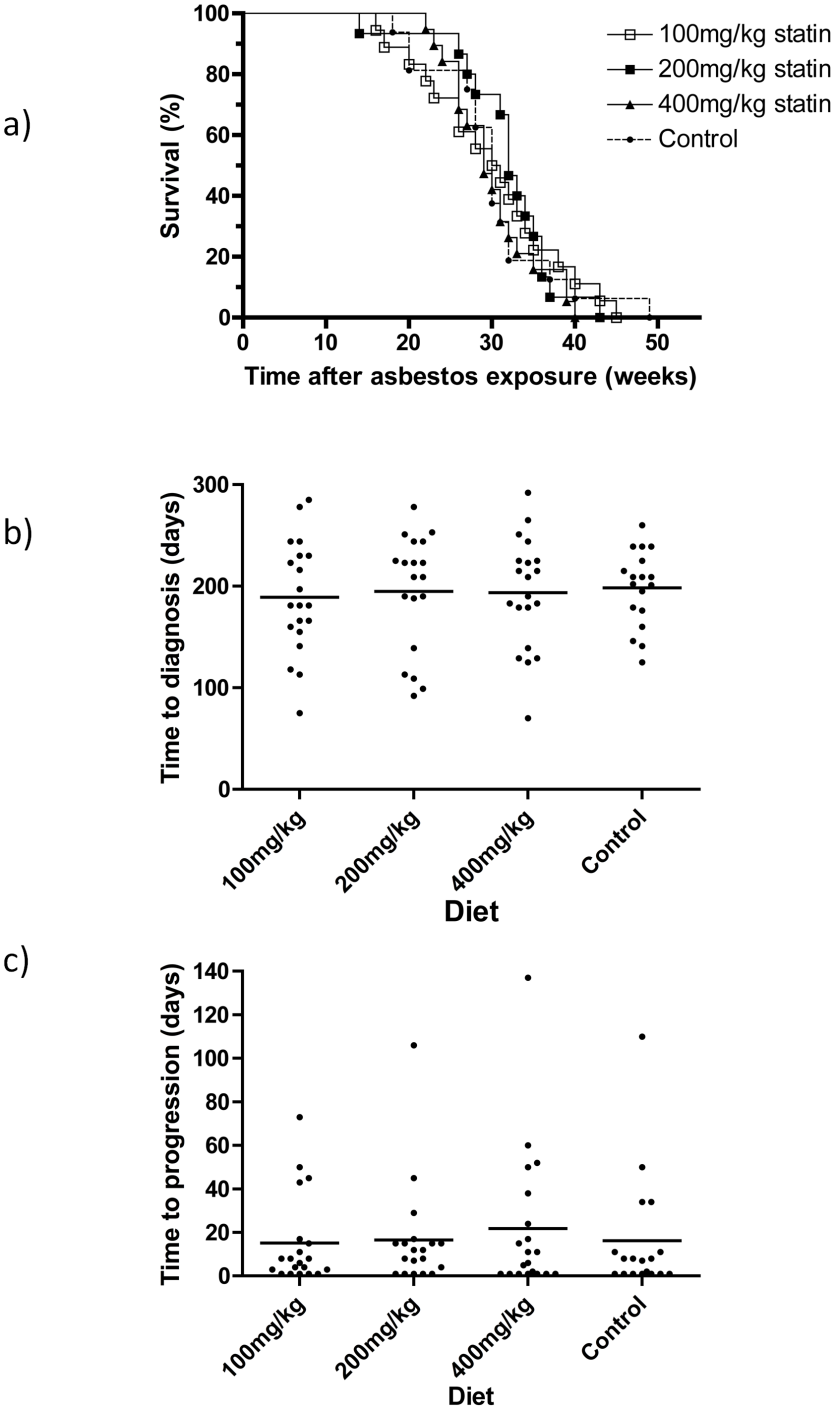
Statin supplementation does not affect mouse survival from asbestos-induced mesothelioma. Diets containing 0 (base diet AIN93), 100, 200 or 400 mg mg/kg of atorvastatin were provided 2 weeks prior to asbestos exposure and for the duration of the experiment. (a) Kaplan Meier survival curve for mice receiving the test diets or base diet (n = 20, log rank test for survival, p = 0.84). (b) Latency time to first diagnosis. One-way analysis of variance across all groups: p = 0.96. (c) Disease progression, defined as survival after diagnosis, one-way analysis of variance across all groups: p = 0.87.

Provision of atorvastatin in mouse diets from 2 weeks prior to disease induction at a range of doses did not alter incidence of or survival with mesothelioma (Fig 1a, p = 0.84). The latency from asbestos exposure to first diagnosis of disease was not significantly different between groups (Fig 1b) and nor was disease progression from first diagnosis (Fig 1c). Thus, in this model, continued use of low to high levels of statins did not affect the rate of mesothelioma development or tumour growth rate once a tumour had arisen.

### Characteristics of the Wittenoom cohort

Participants reporting taking statin or statin in combination with NSAIDs or COX-2i were significantly older than those who did not (p<0.001, Table 1). Use of a statin alone was not different between genders, but reported use of a combination of the drugs was less frequent in females than in males (p<0.001, Table 1). Overweight and obese people reported using a statin or a combination of the drugs more frequently than people with a normal body mass index (BMI). Use of statins was not consistently related to smoking habits. Those subjects working directly in the mines reported significantly higher statin use (alone or in combination with NSAIDs or COX-2i), compared to those who resided in the town but did not work in the mines. However, there was no correlation between the cumulative asbestos exposure and statin use (Table 1).

**Table 1:** Demographic and exposure characteristics of the Wittenoom cohort.

Variable	Whole cohortn = 1754	Statins alone n = 154	NSAIDS/COX-2i alone n = 402	Statins and NSAID or COX-2i n = 400	None n = 798	p value
**Age (at questionnaire)**						<0.001
Yrs, mean (SD)	58.75 (12.13)	62.5 (8.6)	59.6 (10.6)	66.0 (8.3)	54.0 (12.8)	
**Sex, n (%)**						<0.001
male	1235 (70.4)	110 (71.4)	288 (71.6)	316 (79.0)	521 (65.3)	
female	519 (29.6)	44 (28.6)	114 (28.4)	84 (21.0)	277 (34.7)	
**BMI, n(%)**						<0.001
<25, (normal)	343 (19.6)	19 (12.3)	74 (18.4)	53 (13.3)	197 (24.7)	
25–29.9 (overweight)	689 (39.3)	74 (48.1)	159 (39.6)	171 (42.8)	285 (35.7)	
>30 (obese)	468 (26.7)	50 (32.5)	104 (25.9)	145 (36.3)	169 (21.2)	
**Smoking, n(%)**						<0.001
never	573 (32.7)	54 (35.1)	113 (28.1)	124 (31.0)	282 (35.3)	
past	804 (45.8)	71 (46.1)	219 (54.5)	205 (51.3)	309 (38.7)	
current	373 (21.3)	29 (18.8)	70 (17.4)	69 (17.3)	205 (25.7)	
**Occupation, n(%)**						<0.001
workers	1011 (57.6)	95 (61.7)	237 (59.0)	269 (67.3)	410 (51.4)	
Ex-residents	743 (42.4)	59 (38.3)	165 (41.0)	131 (32.8)	388 (48.6)	
**Cumulative asbestos exposure (f/ml.y)**						0.247
<4.85(p50), n(%)	823 (46.9)	74 (48.1)	168 (41.8)	186 (46.5)	395 (49.5)	
4.85–12.98 (p50–p75)	422 (24.1)	32 (20.8)	105 (26.1)	93 (23.3)	192 (24.1)	
>12.98	442 (25.2)	41 (26.6)	114 (28.4)	102 (25.5)	185 (23.2)	

*p-values obtained using Pearson chi-square for categorical variables and one-way analysis of variance for continuous variables (age) across the 4 groups. F/ml.y: fibre per millilitre.year.

### Statin use in the asbestos exposed Wittenoom subjects

554 people reported taking statins, with simvastatin and atorvostatin being most frequently used (Table 2). 346 people reported taking statins plus any NSAID and 54 reported using statin with a COX-2i. 1,200 people reported not using statin and 798 reported using none of these medications.

**Table 2:** Statin and NSAID usage.

Drug name	Number of people using drug	Percent of cohort using drug (n = 1,754)
Simvastatin	225	12.8%
Atorvastatin	200	11.4%
Pravastatin	85	4.8%
Rosuvastatin	32	1.8%
Fluvastatin	11	0.6%
Cerivastatin	1	0.06%
No statins	1,200	68%
Any statin alone	154	8.8%
Any statin plus any NSAID	346	20%
Any statin plus any COX-2i	54	37.7%
Statin, plus NSAID or COX2i	400	22.8%
Statin, NSAID and COX2i	0	0%
No NSAID, COX2i or statin	798	45.5%

### Statin use is not associated with an altered risk of mesothelioma

**Figure 2:**
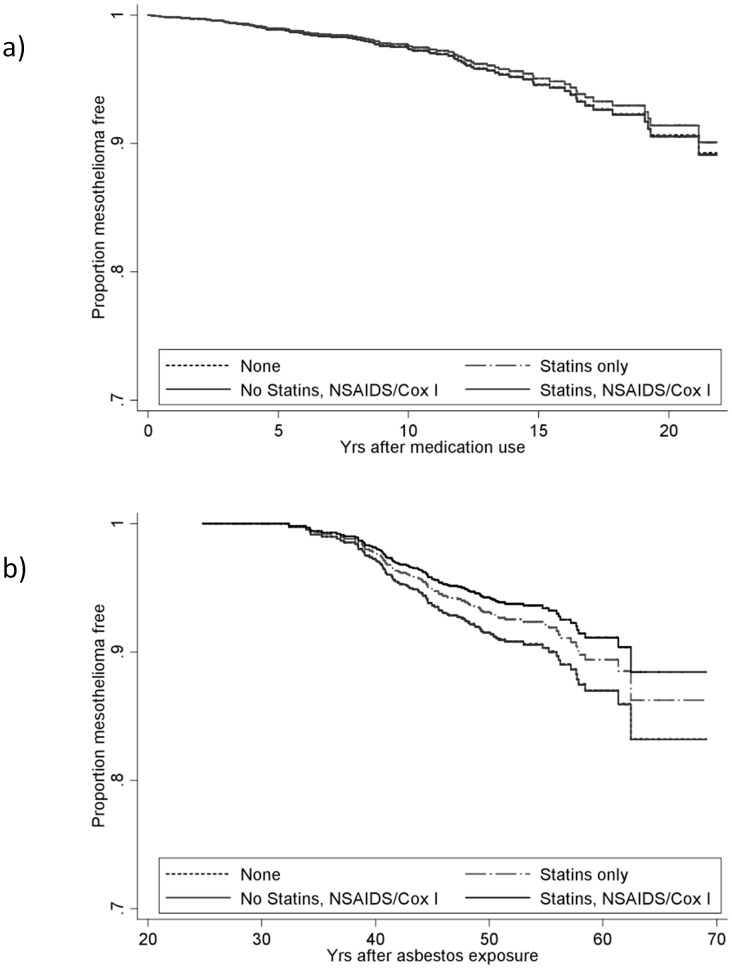
a) Survival from mesothelioma in asbestos-exposed people was not affected by use of statins, COX-2 inhibitors or NSAIDs alone or in combinations indicated in the graph. Survival adjusted for age, sex, BMI, smoking and intensity of asbestos exposure. b) Survival with a diagnosis of mesothelioma from the date of first known asbestos exposure was unaffected by use of statins, COX-2 inhibitors or NSAIDs taken alone or in combination. Adjusted for age, sex, BMI and smoking.

At the end of study date (June 2012) mesothelioma had been diagnosed in 107 people. Participants taking statins alone were no less likely to develop mesothelioma than those not taking the medication (adjusted HR 1.01, 95% CI = 0.44, 2.29; p = 0.99) and nor were those taking statins in combination with NSAIDs or COX-2i (HR 0.91; 95%CI = 0.50–1.66, p = 0.77; Table 3 and Figure 2a)

**Table 3:** Association of medication use and development of mesothelioma in the Wittenoom cohort.

Medication used	No mesothelioma n (%)	Mesothelioma n (%)	Unadjusted HR (95%CI) p-value	Adjusted* since start of study HR (95%CI) p-value	Adjusted** since first asbestos exposure HR (95%CI) p-value
**Neither drug**	747 (93.6)	51 (6.4)	[1]	[1]	[1]
**Statins alone**	146 (94.8)	8 (5.2)	1.19 (0.56,2.52) 0.65	1.01 (0.44,2.29) 0.99	0.81 (0.35,1.83) 0.61
**No statin plus any NSAIDS or COX-2i**	375 (93.3)	27 (6.7)	1.00 (0.63,1.60) 0.99	1.01 (0.61,1.67) 0.97	1.00 (0.61,1.66) 0.99
**Both**	379 (94.8)	21 (5.3)	1.17 (0.70,1.96) 0.55	0.91 (0.50,1.66) 0.77	0.67 (0.37,1.22) 0.19

*Adjusted for age, bmi, sex, smoking status and asbestos exposure category (worker or ex-resident at Wittenoom).

**Additional adjustment for time since asbestos exposure.

Survival from the date of first exposure was also not different for the statin users compared to non-users (HR, 0.81, 95%CI: 0.35–1.83, p = 0.61. Those reporting use of statin plus any NSAID or COX-2i had a lower hazard ratio of 0.67 (95%CI 0.37–1.22), however this was not significant (p = 0.19; Table 3, Figure 2b).

## Discussion

Despite a sound biological rationale for our hypothesis, we found that the use of statins did not alter the rate of development or incidence of mesothelioma in asbestos-exposed mice or people. An analysis of data from the Wittenoom cohort of asbestos-exposed individuals who have been closely monitored for decades found no link between mesothelioma risk and reported use of statins or statins plus either a NSAID or a COX-2i. Similarly, low, medium or high doses of atorvastatin provided to mice from two weeks prior to disease induction with asbestos did not have a significant effect on survival, latency, rate of tumor progression, nor on macroscopic tumor burden.

The MexTAg asbestos-induced model and the Wittenoom cohort of asbestos-exposed individuals are appropriate companion studies because in both cases exposure was to the same type of asbestos, crocidolite, which is considered as more carcinogenic than the more common form, chrysotile [24]. Furthermore, MexTAg tumor pathogenesis is similar to human mesothelioma in terms of many factors including histology, latency, inflammatory response, localization and the response to cytotoxic chemotherapy [12].

In the mouse study the statin was provided starting two weeks prior to asbestos exposure to give maximum chance of detecting an effect. It was continued throughout the experiment, inclusive of the latency and disease progression periods. By comparison, the questionnaire upon which the use of statins and other drugs in the human cohort is based was conducted 24 years after the closure of the Wittenoom mine. Thus, given that human mesothelioma development occurs 40+ years after exposure to asbestos, these data indicate that the drug usage occurred within the latency period; however usage was not restricted to that time. As statins are predominantly administered on a long-term basis to lower cholesterol levels and treat cardiovascular disease, the individuals who reported usage in 1990, are most likely to have continued to do so for the duration of the study. At the time of the questionnaire, statins were new drugs; the first listing of lovastatin was in 1987 and clinical use began in the mid to late 1980s. Statin use in Australia has since escalated due to wider clinical availability [25]. Together with the fact that statin use correlates with age, the number of people taking statins since the start of the questionnaire is anticipated to have increased. This could result in misclassification bias, as some individuals in the control group may have an undisclosed subsequent statin use during the later latency phase. This is a limitation of the data, and as the adjusted hazard ratio between users and non-users was 1.01 (95%CI 0.44–2.29), it is feasible this could significantly affect the results. However, a recent review of multiple randomised controlled trials found no preventative benefit for statins, so it is more likely that this lack of benefit in the prevention of mesothelioma correct. [15].

Within the human cohort there is a diverse genetic background and variation in environmental factors such as lifestyle, diet, smoking, age, as well as levels of asbestos exposure. This is reflected in the variance in latency and survival times [26]. In contrast, in the mouse experiments, these factors are consistent across all study groups, i.e. the mice were genetically identical, lived under the same conditions, had the same base feed and the amount of asbestos received was equivalent. This makes the mouse model an ideal system to investigate the potential of statins to prevent asbestos-induced mesothelioma, and supports the validity of the epidemiological data.

There is excellent concordance in results from our studies of mice and humans not only in this study, but also in our previous work investigating potential preventive agents for mesothelioma. Our two previous investigations addressed vitamin A and non-steroidal anti-inflammatory drug usage and mesothelioma incidence [9], [11]. In the vitamin A trials in both the Wittenoom cohort and the MexTAg mice, the supplementation level was fixed and controlled, and both trials demonstrated no benefit in vitamin A supplementation, despite a strong rationale for a preventative effect. This suggests that comparisons between these sets of mouse and human data are valid. Because we are able to induce mouse mesotheliomas using the same carcinogen as in human mesothelioma, with much the same pathological changes and clinical behaviour, we have an ideal situation to study preventative and therapeutic agents. Such a situation is very rare in the field of animal models of cancer.

There are many examples of negative randomised controlled trials of putative cancer prevention strategies, which were not concordant with previous animal model studies. In some instances a detrimental effect has led to early termination of human clinical trials [27], [28]. However, the key difference between these studies and the one we describe here is that in most murine models the identical carcinogen that causes the human cancer is unable to induce an equivalent mouse cancer, either because the carcinogen is unknown or induces a different tumour or is non-carcinogenic in animals.

The potential benefits from statin use are more complex than might be expected from lowering cholesterol levels alone [29]. Statins have a pleiotropic effect on a number of key pathways, many of which have a role in cancer development, including cell proliferation, angiogenesis, apoptosis, metastasis and inflammation: mechanisms that are relevant to mesothelioma development [30]. For example, statins effectively induce apoptosis and enhance chemosensitivity in mesothelioma cell lines [19], [20]. However, while there have been many proposals for a preventative action of statins, particularly in cohort studies and in vitro cancer cell line data, it is noted that many of these investigations contain caveats [30]. A lack of accord amongst cancer prevention studies in general is common [16]. In the case of statins, this could in part be because the data has mostly arisen from trials that were designed to investigate statin use in the treatment of cardiovascular disease; thus cancer prevention was explored in secondary analyses. In complete conflict, low cholesterol has even been associated with a higher cancer incidence [31]. A recent and comprehensive meta-analysis of randomised controlled trials proposes a lack of effect for statins [15]. Thus, this investigation is in accordance with the findings for the majority of studies.

In the human cohort there was a non-significant trend towards a benefit for combined use of statins and NSAIDs or COX-2i, suggesting the potential for concurrent use of multiple preventative agents to target inhibition of inflammation and other tumorigenic cellular mechanisms. Similarly, people using both NSAIDs and COX-2i compared to use of either drug type individually, also had a trend towards a lower risk of mesothelioma development, albeit non-significant [13]. The number of people using both types of drugs was very small. Thus, despite a hazard ratio that implied a benefit, it did not reach statistical significance. This combinatorial approach could be followed up in the mouse model, but given no effect with the single agents, and the minor improvement in survival associated with multiple drug use in the human cohort, it unlikely that a benefit would be detectable unless very large numbers of mice were used. Nevertheless a combinatorial approach to cancer prevention and more specifically use of statins together with NSAIDs is under investigation [32] and outcomes seem to be dependent on cancer type [33], [34]. While this combination was not found to be inhibitory for mesothelioma development in this investigation, these drugs, which have a strong biological rationale for use in mesothelioma, could potentially be useful in combination with chemotherapy. Combining chemoprevention drugs with cytotoxic chemotherapies is being investigated for other cancers [35] and statins did enhance the efficacy of doxorubicin in mesothelioma cell lines [20].

In conclusion, although statins can induce apoptosis in mesothelioma cells [19] and epidemiological data has linked their use to a lower incidence of cancer, our data show that they do not alter the incidence of mesothelioma in asbestos exposed mice or humans. The long latency between asbestos exposure and presentation of mesothelioma suggests multiple events occur during carcinogenesis and more understanding of this could lead to a more informed approach to cancer prevention. Many people have a known exposure to asbestos and thus any effective prevention agent would immediately benefit this population. The MexTAg model is a good system for testing potential prevention agents because the disease is pathogenically the same as in humans and induced by the same carcinogen, with the distinct advantage that the mice have fewer variables to confound the data. Thus, although statin use did not prevent mesothelioma development, this approach of studying preventative agents in a uniquely relevant animal model plus a relevant patient cohort offers the prospect that other preventative agents, or combinations of such agents, could be evaluated.
